# The role of adrenal venous sampling (AVS) in primary bilateral macronodular adrenocortical hyperplasia (PBMAH): a study of 16 patients

**DOI:** 10.1007/s12020-022-03020-z

**Published:** 2022-03-10

**Authors:** German Rubinstein, Andrea Osswald, Leah Theresa Braun, Frederick Vogel, Matthias Kroiss, Stefan Pilz, Sinan Deniz, Laura Aigner, Thomas Knösel, Jérôme Bertherat, Lucas Bouys, Roland Ladurner, Anna Riester, Martin Bidlingmaier, Felix Beuschlein, Martin Reincke

**Affiliations:** 1 Medizinische Klinik und Poliklinik IV, Klinikum der Universität München, München, Germany; 2 Department of Internal Medicine I, Division of Endocrinology and Diabetes, University of Würzburg, University Hospital Würzburg, Würzburg, Germany; 3 Division of Endocrinology and Diabetology, Department of Internal Medicine, Medical University of Graz, Graz, Austria; 4 Klinik und Poliklinik für Radiologie, Klinikum der Universität München, München, Germany; 5 Department for Diagnostic and Interventional Radiology and Neuroradiology, Klinikverbund Allgäu, Kempten, Germany; 6 Pathologisches Institut, Klinikum der Universität München, München, Deutschland; 7 Université de Paris, 75006 Paris, France; 8 Department of Endocrinology, Center for Rare Adrenal Diseases, Assistance Publique-Hôpitaux de Paris, Hôpital Cochin, 75014 Paris, France; 9 Klinik für Allgemeine, Unfall- und Wiederherstellungschirurgie, Campus Innenstadt, Klinikum der Universität München, München, Germany; 10 Klinik für Endokrinologie, Diabetologie und Klinische Ernährung, Universitätsspital Zürich (USZ) and Universität Zürich (UZH), Zürich, Switzerland

**Keywords:** Cortisol, Aldosterone, Hypercortisolism, Cushing’s syndrome, Adrenal cortex

## Abstract

**Objective:**

Primary bilateral macronodular adrenocortical hyperplasia (PBMAH) is a rare cause of ACTH-independent Cushing’s syndrome. Current guidelines recommend bilateral adrenalectomy for PBMAH, but several studies showed clinical effectiveness of unilateral adrenalectomy despite bilateral disease in selected patients. Our aim was to evaluate the gain of information which can be obtained through adrenal venous sampling (AVS) based cortisol lateralization ratios for guidance of unilateral adrenalectomy.

**Design:**

We performed a retrospective analysis of 16 patients with PBMAH and clinical overt cortisol secretion in three centers

**Methods:**

Selectivity of adrenal vein sampling during AVS was defined as a gradient of cortisol or a reference adrenal hormone ≥2.0 between adrenal and peripheral vein. Lateralization was assumed if the dominant to non-dominant ratio of cortisol to reference hormone was ≥4.0.

**Results:**

AVS was technically successful in all patients based on absolute cortisol levels and in 13 of 16 patients (81%) based on reference hormone levels. Lateralization was documented in 8 of 16 patients. In patients with lateralization, in 5 of 8 cases this occurred toward morphologically larger adrenals, while in 3 patients lateralization was present in bilaterally identical adrenals. The combined volume of adrenals correlated positively with urinary free cortisol, suggesting that adrenal size is the dominant determinant of cortisol secretion.

**Conclusions:**

In this study the gain of information through AVS for unilateral adrenalectomy was limited in patients with PBMAH and marked adrenal asymmetry.

## Introduction

Primary bilateral macronodular adrenocortical hyperplasia (PBMAH) is a rare cause of ACTH-independent Cushing’s syndrome. It is usually identified by adrenal imaging demonstrating bilateral enlarged adrenal masses (symmetric and asymmetric) [1] composed of multiple bilateral macronodules (>10 mm) with hyperplasia and/or internodular atrophy. Rarely, the PBMAH phenotype is associated with primary aldosteronism and hyperandrogenism. The prevalence of PBMAH is probably underestimated, as bilateral adrenal masses are increasingly identified through imaging performed for reasons unrelated to adrenal disease [2]. Along current guidelines for such incidentalomas assessment of endocrine activity is required [3]. The clinical phenotype of Cushing’s syndrome due to PBMAH is usually mild [4]. The optimal treatment for PBMAH with endocrine activity is the subject of ongoing debate. According to the guidelines of the Endocrine Society, first-line treatment of PBMAH with overt Cushing’s syndrome should be bilateral adrenalectomy with consecutive remission and lifelong requirement for steroid replacement therapy [5]. More recently, several studies indicated the effectiveness of unilateral adrenalectomy in bilateral disease [6–11]. Our group has analyzed the long-term outcome after unilateral adrenalectomy for PBMAH and showed that unilateral adrenalectomy leads to clinical remission and a lower incidence of adrenal crisis [12]. It also appeared from this publication that some patients may have persistent hypercortisolism to a clinically relevant degree and potentially higher mortality. However, this has been questioned [13].

The decision of which adrenal to be removed in PBMAH, is generally based on adrenal size. This assumes that a larger adrenal mass causes higher glucocorticoid output. However in endocrinology, morphology does not necessarily predict function. Further methods to evaluate potential lateralization of cortisol production included functional radiotracer imaging to evaluate the adrenal uptake of ^131^I-methylnorcholesterol [7], [14–17], but ^131^I-methylnorcholesterol is no longer available in most countries. Another approach is adrenal venous sampling (AVS) which is commonly used in the diagnostic work-up for primary aldosteronism [18]. AVS might be useful in evaluating the predominant side of cortisol production and may guide the decision for unilateral adrenalectomy in bilateral disease. Four studies with different methods and definitions have evaluated AVS in the context of autonomous cortisol secretion and uni- or bilateral adrenal incidentalomas in small patient groups [17, 19–21]. In addition, several case reports addressed this topic [22–29]. In contrast, patients with PBMAH have not been systematically evaluated. Here we report our experience with AVS in 16 patients with PBMAH from three centers. The aim of this study was to define the relevant clinical gain of information through AVS to identify the hormonally dominant side.

## Methods

### Patients

We performed a retrospective analysis of patients with primary adrenal Cushing’s syndrome and bilateral masses suspicious for PBMAH who underwent AVS to identify the hormonally dominant side of cortisol production. Patients were treated at the endocrine tertiary care centers of the Klinikum der Universität München, Ludwig-Maximilians-Universität Munich, Germany (12 patients), the University hospital of Würzburg, Germany (2 patients) and the University hospital of the University Graz, Austria (2 patients) between 2006 and 2020. Thereby, a total of 16 patients were included. Referral to the centers was triggered by clinical suspicion of Cushing’s syndrome (*N* = 11) or due to incidental detection of bilateral adrenal masses (*N* = 5). Patients underwent evaluation for Cushing’s syndrome following current guidelines [5]. The German Cushing’s Registry was approved by the LMU ethics committee (Project number: 152 -10), and all patients gave written informed consent.

### Adrenal venous sampling (AVS)

We performed AVS in patients with bilateral adrenal masses based on an individual decision of the treating endocrinologists (until 2012) or based on a multidisciplinary endocrine board decision (since 2012) to evaluate and identify the predominant side of cortisol production. Main criteria to proceed with AVS were radiological asymmetry and patient’s willingness to undergo unilateral adrenalectomy.

Peripheral blood samples were collected simultaneously with each of the selective blood samples. AVS was performed without concomitant infusion of ACTH or dexamethasone suppression.

In addition to cortisol, further adrenocortical and –medullary hormones were measured concomitantly as “reference hormones” to verify catheter placement in the adrenal veins and to adjust for dilution. Throughout the 14 years of observation period different reference hormones were used. The choice was based on the technical capability, the state of science and knowledge about PBMAH at that time, and the center’s experience derived from AVS for subtyping of primary aldosteronism. The following hormones were used alone or in combination: Aldosterone (*n* = 13), metanephrine and normetanephrine (*n* = 6), DHEA-S (*n* = 4), or androstenedione (*n* = 2).

For further calculations, the result of one basal sample was used, as the results of additional samples, if available, were synonymously. We post hoc defined successful adrenal vein sampling as a selectivity index (SI) ≥ 2.0: absolute cortisol concentration or “reference hormone” concentration in the adrenal veins at least twice as high than the concentration in the simultaneously drawn peripheral sample. Lateralization of cortisol production was evaluated by the absolute cortisol ratios or the ratio of the cortisol to “reference hormone” ratio from both sides:

$${{{\mathrm{Lateralization}}}}\,{{{\mathrm{index}}}}\,\left( {{{{\mathrm{LI}}}}} \right) = \frac{{\left[ {{{{\mathrm{Cortisol}}}}/{{{\mathrm{reference}}}}\,{{{\mathrm{hormone}}}}} \right]{{{\mathrm{right}}}}\,{{{\mathrm{or}}}}\,{{{\mathrm{left}}}}}}{{\left[ {{{{\mathrm{Cortisol}}}}/{{{\mathrm{reference}}}}\,{{{\mathrm{hormone}}}}} \right]{{{\mathrm{left}}}}\,{{{\mathrm{or}}}}\,{{{\mathrm{right}}}}}}$$

As no consensus on the definition of lateralization in this context exists, the AVS was interpreted in analogy to primary aldosteronism. A predominant cortisol production was usually assumed with a LI ≥ 4 [30]. When the concentrations of the measured hormones were below the lower limit of quantification of the assay, half of the lower limit of quantification was used for calculation. For measured concentrations above the upper limit of quantification, the upper limit of quantification was used for calculation.

### Biochemical and genetic assessment and assays

Biochemical and genetic assessment and assays are described extensively in the online supplement [31].

### Adrenal volume

Adrenal volume was measured three-dimensionally based on the approach of a cuboid by width* depth*height in cm^3^. These measurements were performed by a single radiologist (L.A.). Asymmetry in adrenal size was defined by a difference of more than 30% in volume. In two patients only axial planes were available. Therefore, in these two patients adrenal size was assessed by the maximum diameter in cm and asymmetry defined by a difference of more than 30%.

### Treatment

Recommendation for unilateral or bilateral adrenalectomy was based on the severity of Cushing’s syndrome (clinically and biochemically), the results of radiologic studies and AVS results. Remission after surgery was defined clinically by improvement of catabolic or metabolic features of CS (“clinical remission”) and biochemically by postoperative adrenal insufficiency requiring replacement therapy and/or normalization of all three screening tests for hypercortisolism (“complete biochemical remission”). Partial biochemical remission referred to normalized 24 h urinary free cortisol (UFC), but abnormal 1-mg dexamethasone suppression test (1.8–5 µg/dl) and late night salivary cortisol; biochemical persistence was defined by three abnormal screening tests for hypercortisolism. After surgery the diagnosis of PBMAH was confirmed by an experienced pathologist (T.K.) by the presence of multiple macronodules (>1 cm) and diffuse hyperplasia or atrophy on adrenal specimens.

### Statistics

Pearson’s correlation coefficients (R) were calculated for correlations of adrenal size with overall/bilateral biochemical activity measured as 24 h UFC. IBM SPSS Statistics (version 21.0, IBM North America) was used for statistical analysis and calculation of Pearson’s correlation coefficients and p-values. MS Excel (version 2013) was used to create scatter graphs.

## Results

Sixteen patients were retrospectively analyzed (15 female, 1 male, mean age at diagnosis: 59 ± 9 years). Fourteen patients had clinically overt Cushing’s syndrome. Two patients had mild Cushing’s syndrome with abnormal screening tests and metabolic features, but no catabolic features (e.g., myopathy, skin atrophy, osteoporosis) of Cushing’s syndrome. The presentation of each patient is summarized in Table 1 in the online supplement [31]. Genetic testing for *ARMC5* was performed in 13 patients and revealed single nucleotide variants in 6 patients.

### Adrenal size

Mean adrenal volume was 76.5 ml, ranging from 9.5 to 852 ml. 11 of 16 patients had asymmetric adrenal size, equally distributed on the left (*n* = 6) and right (*n* = 5) adrenal gland. The mean left adrenal volume was 99 ml (range: 11–852 ml, total: 1383 ml) and the mean right adrenal volume was 51 ml (range: 9–137 ml, total: 719 ml).

### Adrenal vein sampling (AVS)

The detailed hormone assessments for each patient are provided in Table 1. Based on the biochemical criteria of absolute cortisol values, all 16 AVS procedures were technically successful. Using the SI calculated on the basis of reference hormones, bilateral catheterization was successful in 13 of 16 (81%) patients, and unilaterally successful in the remaining three patients. The mean SI of all patients on the basis of the absolute cortisol concentrations was 11.3, and that of the reference hormones 17.9.

**Table 1:** Biochemical results from AVS

	Peripheral	Left adrenal	Right adrenal	SI Left	SI Right	LI L/R ratio	LI R/L ratio
Patient 1							
Cortisol [µg/dl]	6.4	28.1	27.6	4.4	4.3	1.0	
Aldosterone [ng/l]	78.7	342.3	241.8	4.3	3.1		**1.4**
Metanephrine [pg/ml]							
Normetanephrine [pg/ml]							
DHEA-S [µg/ml]	2.6	2.9	3.1	1.1	1.2	1.1	
Androstenedione [ng/ml]							
Patient 2							
Cortisol [µg/dl]	15.1	106	35.8	7.0	2.4	3.0	
Aldosterone [ng/l]							
Metanephrine [pg/ml]							
Normetanephrine [pg/ml]							
DHEA-S [µg/ml]	<0.1	<0.1	0.2	1	4	**11.8**	
Androstenedione [ng/ml]							
Patient 3							
Cortisol [µg/dl]	16.8	44.3	32.8	2.6	2.0	1.4	
Aldosterone [ng/l]	<35	58.2	83.2	3.3	4.8	**1.9**	
Metanephrine [pg/ml]							
Normetanephrine [pg/ml]							
DHEA-S [µg/ml]							
Androstenedione [ng/ml]							
Patient 4							
Cortisol [µg/dl]	22.9	127.2	125.7	5.6	5.5	1.0	
Aldosterone [ng/l]	<35	110	116	6.3	6.6	**1.1**	
Metanephrine [pg/ml]	<36	87	365	4.8	20.3	4.2	
Normetanephrine [pg/ml]	<48	52	100	2.2	4.2	1.9	
DHEA-S [µg/ml]							
Androstenedione [ng/ml]							
Patient 5							
Cortisol [µg/dl]	12.9	37.7	271.9	2.9	21.1		7.2
Aldosterone [ng/l]	<35	1720	272	98.3	15.5		**45.6**
Metanephrine [pg/ml]							
Normetanephrine [pg/ml]							
DHEA-S [µg/ml]							
Androstenedione [ng/ml]							
Patient 6							
Cortisol [µg/dl]	10.8	512	616.8	47.4	57.1		1.2
Aldosterone [ng/l]							
Metanephrine [pg/ml]	<36	5987	2288	332.6	127.1		**3.2**
Normetanephrine [pg/ml]	66	1617	568	24.5	8.6		3.4
DHEA-S [µg/ml]							
Androstenedione [ng/ml]							
Patient 7							
Cortisol [µg/dl]	8.6	38.9	216.9	4.5	25.2		5.6
Aldosterone [ng/l]							
Metanephrine [pg/ml]	40	88	2105	2.2	52.6	**4.3**	
Normetanephrine [pg/ml]	<48	<48	552	1.0	23	4.1	
DHEA-S [µg/ml]							
Androstenedione [ng/ml]							
Patient 8							
Cortisol [µg/dl]	10.4	633.5	50.9	60.9	4.9	12.5	
Aldosterone [pg/ml]	239	1840	2190	7.7	9.2	**14.8**	
Metanephrine [pg/ml]	<36	2096	1005	116.4	55.8	6	
Normetanephrine [pg/ml]	<48	601	371	25	15.5	7.7	
DHEA-S [µg/ml]							
Androstenedione [ng/ml]							
Patient 9							
Cortisol [µg/dl]	7.8	179	17	22.9	2.2	10.5	
Aldosterone [ng/l]	<35	179	220	10.2	12.6	**12.9**	
Metanephrine [pg/ml]							
Normetanephrine [pg/ml]							
DHEA-S [µg/ml]							
Androstenedione [ng/ml]							
Patient 10							
Cortisol [µg/dl]	11.7	41.3	271.7	3.5	23.2		**6.6**
Aldosterone [pg/ml]	86	140	357	1.6	4.2		2.6
Metanephrine [pg/ml]							
Normetanephrine [pg/ml]							
DHEA-S [µg/ml]							
Androstenedione [ng/ml]	<0.24	9.4	24.24	78.3	202		2.6
Patient 11							
Cortisol [µg/dl]	14.5	154.8	32.4	10.7	2.2	4.8	
Aldosterone [pg/ml]	94	1270	329	13.5	3.5	1.2	
Metanephrine [pg/ml]							
Normetanephrine [pg/ml]							
DHEA-S [µg/ml]							
Androstenedione [ng/ml]	0.66	7.94	7.25	12.0	11.0	**4.4**	
Patient 12							
Cortisol [µg/dl]	9.0	23.7	39.7	2.6	4.4		1.7
Aldosterone [pg/ml]	52	239	277	4.6	5.3		**1.4**
Metanephrine [pg/ml]							
Normetanephrine [pg/ml]							
DHEA-S [µg/ml]							
Androstenedione [ng/ml]							
Patient 13							
Cortisol [µg/dl]	10.6	35.6	22.0	3.4	2.1	1.6	
Aldosterone [ng/l]	37	63	315	1.7	8.5	**8.1**	
Metanephrine [pg/ml]							
Normetanephrine [pg/ml]							
DHEA-S [µg/ml]							
Androstenedione [ng/ml]							
Patient 14							
Cortisol [µg/dl]	12.4	74.3	46.9	6.0	3.8	1.6	
Aldosterone [ng/l]	37	1415	420	38.2	11.4		**2.1**
Metanephrine [pg/ml]							
Normetanephrine [pg/ml]							
DHEA-S [µg/ml]							
Androstenedione [ng/ml]							
Patient 15							
Cortisol [µg/dl]	8.5	28.8	56.5	3.4	6.6		2.0
Aldosterone [ng/l]	47	1008	>1320	21.4	28.1		**1.5**
Metanephrine [pg/ml]	<28	2410	1770	172	126		2.7
Normetanephrine [pg/ml]	57	663	503	11.6	8.8		2.6
DHEA-S [µg/ml]	0.18	0.23	0.24	1.3	1.3		1.9
Androstenedione [ng/ml]							
Patient 16							
Cortisol [µg/dl]	16.6	48.8	>75	2.9	4.5		1.5
Aldosterone [ng/l]	<37	311	667	16.8	36.1	**1.4**	
Metanephrine [pg/ml]	995	47	47	0.0	0.0		1.5
Normetanephrine [pg/ml]	210	68	40	0.3	0.2		2.6
DHEA-S [µg/ml]	0.04	0.06	0.11	1.5	2.8	1.2	
Androstenedione [ng/ml]							

Lower limits of quantification: Cortisol 2 µg/dl, Aldosterone: 35 ng/ml, Metanephrine: 36 pg/ml, Normetanephrine: 48 pg/ml, DHEA-S 0.1 µg/ml, Androstenedione 0.24 ng/ml. When the concentration of the measured hormones was below the lower limit of quantification of the assay, half of the lower limit of quantification was used for calculation. Hormone concentrations above the upper limit of quantification were reported as the upper limit of quantification. The LI, which was used for the interpretation of its patient’s AVS, is marked *bold*. The patients’ 4 AVS was considered as bilateral, because the lateralization indices adjusted for aldosterone and normetanephrines were 1.9 and 1.1 (despite the lateralization index adjusted for metanephrines of 4.2)

*SI* Selectivity index, *LI* Lateralization index, *L* Left, *R* Right

Cortisol lateralization indices ≥4.0 were present in 7 of 16 patients (reference hormone based LI) and 6 of 16 (based on absolute cortisol levels). In total, in 8 of 16 patients the AVS was defined as lateralized (7 patients based on reference hormone LI plus 1 patient based on absolute cortisol LI). In 4 of these 8 patients LI’s were contrary for reference hormone based LI and absolute cortisol based LI (Patients 2, 7, 10, 13; Table 1). In patients in whom lateralization was present, it occurred toward the larger adrenal in 5 patients, toward the smaller adrenal in no patient, and toward one side in 3 patients with equally enlarged adrenal. In the latter three patients, AVS showed maximum lateralization indices of 11.8, 14.8 and 4.4 (Patients 2, 8 and 11; Table 1), respectively.

### Treatment and outcome

**Fig. 1:**
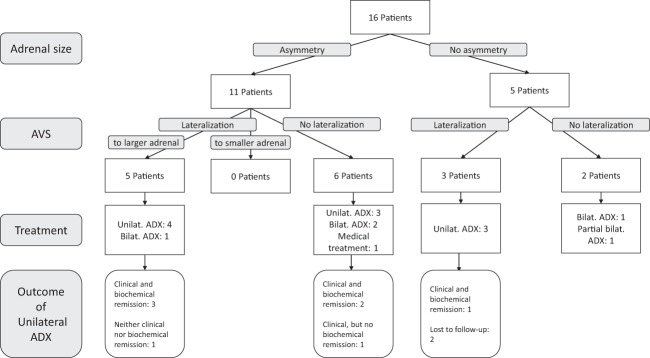
Decision diagram. Definitions of remission: Clinical remission: Improvement of catabolic or metabolic features of CS. Biochemical remission: Adrenal insufficiency and/or normalization of screening tests for hypercortisolism. AVS: Adrenal venous sampling, Unilat: Unilateral, Bilat: Bilateral, ADX: Adrenalectomy

Decision on treatment was based on asymmetry in adrenal size and on lateralization in AVS. Table 2 shows for each patient the interpretation of AVS, the applied surgery and postoperative results (pathology and biochemical assessment). Figure 1 shows the clinical and biochemical outcome separated into patients with morphological asymmetry and with adrenals of equal size.

**Table 2:** Interpretation of AVS, applied surgery, and postoperative results (pathology and biochemical assessment)

Patient No.	Year of AVS	Radiological asymmetry	Interpretation of AVS (Hypercortisolism)	Surgery	*ARMC5* status	Histopathology	Clinical remission	Biochemical remission	Follow-up [months]
Patient 1	2006	L < R	Bilateral	Right ADX	wt	PBMAH	Yes	Complete, since 2015 hypercortisolism	165
Patient 2	2008	L = R	Left-sided	Left ADX	wt	PBMAH	NA	NA	Lost to follow-up
Patient 3	2007	L > R	Bilateral	Left ADX	wt	PBMAH	Yes	Persistence	16
Patient 4	2012	L > R	Bilateral	Bilateral ADX	MUT c.2290C > T, p.R764X	PBMAH	Yes	Complete	95
Patient 5	2010	L < R	Right-sided	Bilateral ADX	wt	PBMAH	Yes	Complete	96
Patient 6	2017	L = R	Bilateral	Partial bilateral ADX (left complete, right ½)	wt	PBMAH	Yes	Complete	32
Patient 7	2017	L > R	Left-sided	Left ADX	NA	PBMAH	Yes	Complete, at last follow-up hypercortisolism	13
Patient 8	2018	L = R	Left-sided	Left ADX	MUT NM_001105247:exon3:c.G646A:p.V216M	PBMAH	NA	NA	Lost to follow-up
Patient 9	2010	L > R	Left-sided	Left ADX	NA	PBMAH	Yes	Complete	106
Patient 10	2019	L < R	Right-sided	Right ADX	wt	PBMAH	No	Persistence -> Treatment with Metyrapone	20
Patient 11	2019	L = R	Left-sided	Left ADX	MUT NM_001105247:exon1:c.237_238insC:p.A80Rfs*23	PBMAH	Yes	Complete	15
Patient 12	2019	L < R	Bilateral	Right ADX	NA	PBMAH	Yes	Complete	21
Patient 13	2016	L > R	Left-sided	Left ADX	MUT c.41T > A = p.Phe14Tyr, rs151069962	PBMAH	Yes	Complete	46
Patient 14	2016	L > R	Bilateral	No surgery, Treatment with Ketoconazole	MUT c.508A > G = p.Ile170Val, rs35923277	Not applicable			34
Patient 15	2018	L < R	Bilateral	Bilateral ADX	wt	Bilateral Adenoma	Yes	Complete	18
Patient 16	2018	L = R	Bilateral	Bilateral ADX	MUT c.1961 G > T, p.Arg654Leu and c.1864 + 73C > T	PBMAH	Yes	Complete	12

Remission (in patients who underwent surgery) was defined clinically (yes/no) and biochemically (complete/partial/persistence)

*AVS* Adrenal venous sampling, *L* Left, *R* Right, *ADX* Adrenalectomy, *PBMAH* Primary Bilateral Macronodular Adrenocortical Hyperplasia, *NA* not available, *Wt* Wildtype

Five patients had both asymmetry in adrenal size and lateralization on AVS (always toward the radiologically larger side). One patient underwent bilateral adrenalectomy with consecutive clinical and biochemical remission (patient 5). Four patients underwent unilateral adrenalectomy: Three patients achieved both clinical and biochemical remission (patients 7, 9, and 13). The remaining patient achieved neither clinical nor biochemical remission and was therefore treated with metyrapone (patient 10).

Six patients had asymmetry in adrenal size, but no lateralization in AVS: One of these patients received medical treatment with ketoconazole (patient 14). Two of these patients underwent bilateral adrenalectomy with consecutive clinical and biochemical remission (patients 4 and 15). The three remaining patients underwent unilateral adrenalectomy of the larger adrenal gland: Two of these patients achieved both clinical and biochemical remission after surgery (patients 1 and 12). The remaining patient (patient 3) achieved clinical, but no biochemical remission.

Three patients had no asymmetry in adrenal size but lateralization in AVS: All three patients underwent unilateral adrenalectomy based on AVS results. Two patients are lost to follow-up (patients 2 and 8) and no postoperative information is available. The other patient had clinical and biochemical remission (patient 11).

Two patients had neither asymmetry in adrenal size nor lateralization in AVS: One of these patients underwent (complete) bilateral adrenalectomy (patient 16) and the other partial bilateral adrenalectomy (left side: complete, right side ½ removal, patient 6). Both patients achieved clinical and biochemical remission.

In summary, outcome of patients with asymmetric enlargement of adrenals, who underwent unilateral adrenalectomy, were similar regardless of lateralization seen in AVS prior to surgery.

### Correlation between urinary free cortisol and adrenal volume

**Fig. 2:**
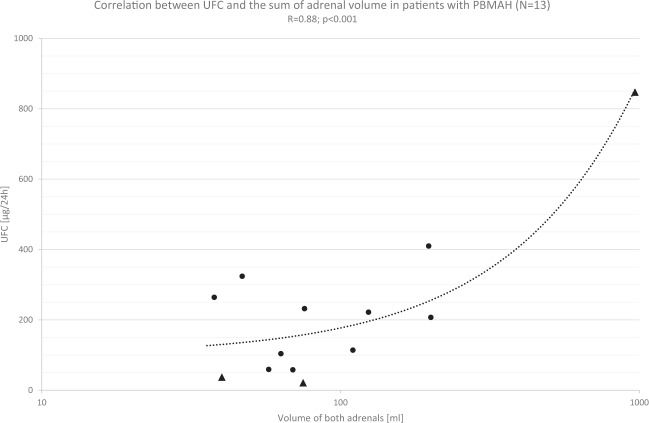
Correlation of 24 h urinary free cortisol (UFC) with the volume of both adrenals from the same patient. The *X*-axis is shown as a logarithmic scale. Triangles represent the patients with *ARMC5* variants (*N* = 3)

Correlation of the 24 h UFC as a marker of overall biochemical activity with the combined bilateral volume from both adrenals from the same patient showed an excellent correlation (Fig. 2: *R* = 0.88, *p* < 0.001, *N* = 13). The relationship was strongly influenced by one case (to the far right in Fig. 2). Without this case, the R coefficient was 0.45 (*p* = 0.143). This indicates that the volume of adrenals in patients with PBMAH may be one of the main determinants of cortisol production.

## Discussion

In this study in PBMAH patients with Cushing’s syndrome we provide evidence that AVS is able to detect lateralization of adrenal cortisol production in roughly 50% of patients. In 5 out of 8 patients, lateralization was observed toward the radiologically larger adrenal gland, while the remaining patients had equally enlarged adrenals. In no case lateralization was found toward the smaller gland. In addition, correlation of adrenal size and 24 h UFC (as a marker of overall biochemical activity) indicates that size of the adrenals may be one of the main determinants of cortisol production. Therefore, we conclude that the gain of information through AVS in the context of Cushing’s syndrome due to PBMAH with marked asymmetry of the adrenals is limited, since AVS does not provide additional information on top of radiological imaging. Importantly, the surgical outcomes were similar in patients without AVS gradients compared to those with gradients (Fig. 1). This conclusion concerns only patients with asymmetric enlargement, since our series was too small for patients with symmetric enlargements to draw conclusions.

AVS is mainly used for the diagnostic work-up in primary aldosteronism. In adrenal incidentalomas with autonomous cortisol production four studies have evaluated the usefulness of AVS, and all of them judge AVS as a helpful diagnostic procedure.

In all of these studies dexamethasone was administered prior to AVS to induce full ACTH suppression. As our patients had ACTH-independent symptomatic Cushing’s syndrome (*n* = 14) or autonomous cortisol production with low or suppressed ACTH levels, dexamethasone was not administered prior to AVS.

One of the main differences in the studies from the literature (Table 3) are the different definitions of correct drainage and lateralization: In some of these studies absolute cortisol concentrations in the adrenal veins were used for interpretation of AVS. At variance, in primary aldosteronism, measurement of a non-target hormone (cortisol) is used in AVS protocols to adjust the target hormone aldosterone, for two reasons: Firstly, hormonal verification of correct drainage is obligatory as drainage of especially the right adrenal vein can be very challenging [32]. Secondly, adjustment for dilution effects of vena cava blood is necessary in order to compare adrenal cortisol concentrations from both sides. In our study 12 of 16 patients had the same interpretation of the AVS comparing absolute cortisol levels with adjusted cortisol lateralization indices. We followed mainly the adjusted cortisol levels. However, the AVS from patient 10 was considered as lateralized because absolute cortisol levels showed almost sevenfold higher levels in the right adrenal vein (compared to a reference hormone based LI of 2.6).

**Table 3:** Literature review

Study	Year	Number of patients	Indication for AVS	Dexa- methasone supression	Reference hormone	Definition of correct drainage	Definition of lateralization
Papakokkinou et al. [19]	2019	10	Bilateral adrenal incidentaloma and hypercortisolism	Yes	Aldosterone (*N* = 10), DHEA-S (*N* = 10), Epinephrine (*N* = 8), Norepinephrine (*N* = 8)	Adrenal to peripheral vein ratio of aldosterone >2	Side to side ratio of Cortisol and reference hormone >2
Ueland et al. [17]	2018	34	Adrenal incidentaloma (uni- and bilateral) and autonomous cortisol secretion	Yes	Metanephrine	Adrenal to peripheral vein ratio of metanephrine >12	Side to side ratio of absolute cortisol >2,3
Acharya et al. [20]	2018	8	Bilateral adrenal Incidentaloma	Study protocol of Young et al. [21]			
Seki et al. [22]	2015	3	Bilateral adrenal masses and ACTH-independent CS	No (but ACTH stimulation)	Aldosterone (for evaluation of PA)	Not defined	Not defined
Young et al. [21]	2007	10	Bilateral adrenal incidentaloma and overt or subclinical CS	Yes	Epinephrine	Epinephrine concentration in adrenal vein exceeding periphery by 100 pg/ml	Side to side ratio of absolute cortisol >2,3
Rubinstein et al. (present study)	2021	16	PBMAH	No	Aldosterone (*N* = 13), Metanephrine (*N* = 6), Normetanephrine (*N* = 6), DHEA-S (*N* = 4), Androstenedione (*N* = 2)	Adrenal to peripheral vein ratio of cortisol or “reference home” >2	Side to side ratio of Cortisol and reference hormone >4

Literature review of studies of AVS performed in the setting of hypercortisolism (with at least three patients). Case reports on single patients were excluded

*CS* Cushing’s syndrome, *PA* Primary aldosteronism

Which hormone to use for defining correct drainage and adjust for dilution is challenging to decide and also the main limitation of our study. Due to the long-lasting time interval of our retrospective analysis and changes in principal investigators during this time the reference hormones have changed over the years. In fact, a potpourri of the adrenal hormones (aldosterone, DHEA-S and androstenedione as androgens and the adrenomedullary hormones metanephrine and normetanephrine) was used for this purpose. During the last years the scientific advances increased significantly, including the discovery of biallelic inactivation of the *ARMC5* gene necessary for the growth of adrenal nodules, paracrine ACTH secretion and the autonomous macronodules inside a PBMAH adrenal gland leading to different endocrine phenotypes [33–36]. Moreover, androgens have a quite long half-time and therefore do not seem to perform superior. The use of DHEA-S as a reference hormone is questionable, as in our study the measured levels of DHEA-S were very low and often close to or below the lower limit of detection. Due to its origin from the medulla and its presumed independency from fluctuations due to stress (in contrast to epinephrine) the use of metanephrine as a reference hormone may be superior. In the setting of primary aldosteronism the measurement of metanephrines has been recommended [37]. With reference to AVS in ACTH-independent Cushing’s syndrome aldosterone itself may also serve as reference hormone, when excess of aldosterone due to PBMAH is excluded.

Most of our patients had clinically overt Cushing’s syndrome and further diagnostic work-up revealed the suspicion of PBMAH. In contrast, in the previous studies mentioned above, AVS was mainly performed in patients with bilateral adrenal incidentaloma and endocrine activity was usually limited and defined as subclinical Cushing’s syndrome or mild autonomous cortisol secretion. A direct comparison is therefore not possible. With respect to PBMAH, lateralization in AVS was mainly to the radiologically larger adrenal gland. We, therefore, argue against routine use of AVS in asymmetric PBMAH. Whether it may be of use in patients with symmetric PBMAH needs to be analyzed in a series of larger size than ours.

### Limitations

Co-secretion of aldosterone was not assessed systematically, therefore interpretation of AVS based on aldosterone-corrected cortisol values could be confounding. Main limitation of our study is the retrospective analysis and the long time span. During this time individual decisions for treatment did not follow a predefined protocol as also medical staff changed. This applies also to the diagnostic use of the AVS and the variety of reference hormones used. Unfortunately, of the small number of patients in this study some patients were lost to follow-up. Therefore, evaluation of the postoperative outcome could not be assessed in all patients. Nevertheless, we think that our data is robust enough to derive our main conclusions from it.

## Supplementary Information

Rubinstein_AVS in PBMAH_supplementary data

## Abbreviations

PBMAH: Primary bilateral macronodular adrenocortical hyperplasia
AVS: Adrenal venous sampling
