# Supplementary material for: The role of adrenal venous sampling (AVS) in primary bilateral macronodular adrenocortical hyperplasia (PBMAH): a study of 16 patients
Source: Endocrine. 2022 Mar 10;76(2):434–45. doi: 10.1007/s12020-022-03020-z (PMC9068666; doi:10.1007/s12020-022-03020-z)
Supplement: Supplementary file 1 — Rubinstein_AVS in PBMAH_supplementary data [file 12020_2022_3020_MOESM1_ESM.pdf]

Supplementary data from:

# The role of Adrenal venous sampling (AVS) in Primary Bilateral Macronodular Adrenocortical Hyperplasia (PBMAH): A study of 16 patients

German Rubinstein, Andrea Osswald, Leah Theresa Braun, Frederick Vogel, Matthias Kroiss, Stefan Pilz, Sinan Deniz, Laura Aigner, Thomas Knösel, Jérôme Bertherat, Lucas Bouys, Roland Ladurner, Anna Riester, Martin Bidlingmaier, Felix Beuschlein, Martin Reincke

The associated article has been submitted to *Endocrine.*

Please cite the original article when using these data.

**Methods:**

***Biochemical and genetic assessment and assays***

Biochemical screening for Cushing's syndrome was performed with all three recommended screening tests: 1-mg dexamethasone suppression test, late-night salivary cortisol and 24h urinary free cortisol measurement. The assays at the study site in Munich are indicated below. The assays at the study sites Würzburg and Graz are indicated in Table 2.

Concentrations of cortisol in saliva were measured by chemiluminescence immuno-assay (IDS, iSYS Analyzer, before June 2016: IBL Cortisol LIA Kitinsert). Urinary free cortisol was measured by chemiluminescence immuno-assay after extraction with dichloromethane (DiaSorin, Liaison, before October 2015: ADVIA Centaur cortisol assay, Siemens Healthcare Diagnostics).

Cortisol in serum was measured with a Solid Phase Antigen linked Technique (DiaSorin Kitinsert Liaison; maximum inter- and intra assay coefficient of variation (CV): 12% and 4%). Measurement of ACTH, aldosterone, DHEA-S and androstenedione was performed with an automated chemiluminescence immuno-assay (DiaSorin, Liaison; maximum inter- and intra

assay CV: ACTH: 15% and 16%; aldosterone: 15% and 6%; DHEA-S: 20% and 9%; androstenedione: 15% and 10%). Before March 2014 aldosterone measurement was performed with a radioimmunoassay “aldosterone Coat-a-Count” (Biermann DPC). Metanephrine and normetanephrine concentrations were determined by the 2-MET Plasma Enzyme-linked Immunosorbent Assay Kit (LDN Labor Diagnostika Nord; maximum inter- and intra assay CV: 29% and 28%). All samples from each AVS were analyzed in one run.

Genetic testing for the ARMC5 mutation status was performed at the Cochin Institute in Paris for the patients from Munich and Würzburg. Until 2019, it was performed as previously described by Sanger sequencing [1]. From 2020, ARMC5 coding sequence and flanking intronic sequences were sequenced from leukocyte DNA using the Ion S5™ XL Next-Generation Sequencing system (Ion Torrent, Thermo Fisher Scientific, USA). All mutations were confirmed twice in two independent experiments. The in silico softwares Polyphen-2 (http://genetics.bwh.harvard.edu/pph2/) and SIFT version 2 (http://sift.jcvi.org/www/SIFT_enst_submit.html) were used to predict the pathogenic potential of the missense variants. The software Mutalyzer (Version 2.0.33; https://mutalyzer.nl/name-checker/) was used to check the sequence variant nomenclature according to Human Genome Variation Society version 2.0.

**Tables:**

**Table 1: Clinical, biochemical and radiological characteristics.** Abbreviations: CS: Cushing’s syndrome; MACS: Mild autonomous Cortisol secretion; F: Female; M: Male; LDDST: Low Dose Dexamethasone Suppression Test (normal range: <1,8µg/dl); UFC: 24h urinary free cortisol measurement (normal range: 50-150µg/24h); LNSLC: late-night salivary cortisol (normal

range: <1,5ng/ml); ACTH: Adrenocorticotropic hormone (normal range: 10-50pg/ml); WxDxH: Width x Depth x Height; Wt: Wildtype; NA: not available.

**Table 2: Biochemical and genetic assays at the study sites Würzburg and Graz.** Abbreviations: PFBHA: 0-(2,3,4,5,6-pentafluorobenzyl) hydroxylamine hydrochloride; MSTFA: methyltrimethylsilyltrifluoracetamide; PCR: polymerase chain reaction.

**Table 1**

| **Patient No.** | **Sex and Age (at diagnosis)** | **Clinical presentation** | **LDDST [µg/dl]** | **UFC [µg/24h]** | **LNSLC [ng/ml]** | **ACTH [pg/ml]** | **Adrenal size on imaging** | | | | **ARMC5 Status** |
|---|---|---|---|---|---|---|---|---|---|---|---|
| | | | | | | | **Left** | | **Right** | | |
| | | | | | | | **WxDxH [cm]** | **Volume [ml]** | **WxDxH [cm]** | **Volume [ml]** | |
| **Patient 1** | F 58 | Overt CS | 7.6 | 410 | 7.9 | 2.5 | 2.8x5.1x4.2 | 60 | 5.5x4.9x5.1 | 137.4 | wt |
| **Patient 2** | F 60 | Overt CS | 5.6 | 169 | 2.3 | 9 | 2.6x5x4.2 | 54.6 | 2.3x4.3x5.6 | 55.4 | wt |
| **Patient 3** | F 56 | Overt CS | 15.1 | 207 | NA | <5 | 5x4.8x5.8 | 139.2 | 3.5x3.9x4.5 | 61.4 | wt |
| **Patient 4** | M 48 | Overt CS | 33.7 | 847 | 10.3 | 3 | 8.2x10.5x9.9 | 852.4 | 4.7x3.9x6.3 | 115.5 | MUT c.2290C>T, p.R764X |
| **Patient 5** | F 58 | Overt CS | 4.1 | 222 | 3.3 | <2 | 2.2x3.4x4 | 29.9 | 3.7x4.8x5.3 | 94.1 | wt |
| **Patient 6** | F 38 | Overt CS | 14.5 | 324 | 5.9 | 3 | 4x1.3x4.4 | 22.9 | 1.4x3.5x4.9 | 24 | wt |
| **Patient 7** | F 72 | Overt CS | 3.1 | 322 | 0.7 | 4 | 3x2.3x4.1 | 28.3 | 1.8x2.1x2.5 | 9.5 | NA |
| **Patient 8** | F 50 | Overt CS | 13.2 | NA | 5.3 | 2 | 2.6x3.3x1.9 | 16.3 | 1.5x3.5x3.7 | 19.4 | MUT NM_001105247:exon3:c. |

| | | | | | | | | | | | |
|---|---|---|---|---|---|---|---|---|---|---|---|
| | | | | | | | | | | | G646A:p.V216M |
| **Patient 9** | F 64 | MACS | 5.6 | 59 | 0.7 | NA | 3.2x2.8x3.7 | 33.2 | 2.4x2.6x3.9 | 24.3 | NA |
| **Patient 10** | F 56 | Overt CS | 8.0 | 104 | 4.1 | 4 | 2.7x2.9x2.8 | 21.9 | 3.5x3.8x3.1 | 41.2 | wt |
| **Patient 11** | F 58 | Overt CS | 4.3 | 151 | 5.6 | <2 | 2.2x3.1 | | 3.1x2.0 | | MUT NM_001105247:exon1:c.237_238insC:p.A80Rfs*23 |
| **Patient 12** | F 60 | Overt CS | 25.1 | 232 | 4.32 | 3 | 3.8x2x1.5 | 11.4 | 5.6x2.5x4.6 | 64.4 | NA |
| **Patient 13** | F 55 | MACS | 5.5 | 21.3 | 3.6 | <5 | 3.8x4.7x3.4 | 60.7 | 2.0x2.3x3.1 | 14.3 | MUT c.41T>A = p.Phe14Tyr, rs151069962 |
| **Patient 14** | F 77 | Overt CS | 8.8 | 36.8 | 1.6 | 6.3 | 3.3x2.6x3.0 | 25.7 | 2.8x1.9x2.7 | 14.4 | MUT c.508A>G = p.Ile170Val, rs35923277 |
| **Patient 15** | F 69 | Overt CS | 17.2 | 58 | 30.0 | <5 | 4.5x2.6x2.2 | 25.7 | 5.4x2.6x3.1 | 43.5 | wt |

| | | | | | | | | | | | |
|---|---|---|---|---|---|---|---|---|---|---|---|
| **Patient 16** | F 62 | Overt CS | 17.4 | 50 | 4.1 | <5 | 2.0x2.2 | | 2.8x1.9 | | MUT c.1961 G>T, p.Arg654Leu and c.1864+73C>T |

**Table 2**

| Assay | Würzburg, Germany | Graz, Austria |
|---|---|---|
| **Cortisol in serum** | Immulite 2000 XPi chemiluminescent immunometric assay (Siemens Healthcare Diagnostics Inc.) | ADVIA Centaur Cortisol-test on a CENTAUR XP (Siemens Healthcare Diagnostics Inc.) |
| **ACTH** | Immulite 2000 XPi chemiluminescent immunometric assay (Siemens Healthcare Diagnostics Inc.) | Immulite 2000 XPi chemiluminescent immunometric assay (Siemens Healthcare Diagnostics Inc.) |
| **24h urinary free cortisol** | Cortisol radioimmunoassay kit (Beckman Coulter) | LC-MS/MS on a HP5-MS column (derivatisation with PFBHA (0-(2,3,4,5,6-pentafluorobenzyl) hydroxylamine hydrochloride) and MSTFA (methyltrimethylsilyltrifluoracetamide) |
| **Cortisol in saliva** | Lumineszenz immunoassay (IBL International GmbH) | Enzyme-linked immunosorbent assay kit (Demeditec Diagnostics) |
| **Aldosterone** | Automated chemiluminescence immunoassay (CLIA, iSYS, Immuno Diagnostic Systems) | Chemiluminescence assay (IDS-iSYS Aldosterone assay; Immunodiagnostic Systems Ltd.) |
| **Metanephrine** | 2-MET Plasma Fast track radioimmunoassay (Labor Diagnostika Nord) | MetCombi Plasma Enzyme-linked immunosorbent assay (DRG Instruments GmBh) |
| **Normetanephrine** | 2-MET Plasma Fast track radioimmunoassay (Labor Diagnostika Nord) | MetCombi Plasma Enzyme-linked immunosorbent assay (DRG Instruments GmBh) |
| **DHEA-S** | Immulite 2000 XPi chemiluminescent immunometric assay (Siemens Healthcare Diagnostics Inc.) | Enzyme-linked immunosorbent assay (Labor Diagnostika Nord) |
| **Androstendione** | Immulite 2000 XPi chemiluminescent immunometric assay (Siemens Healthcare Diagnostics Inc.) | Enzyme-linked immunosorbent assay (Siemens Healthcare Diagnostics Products Ltd.) |
| **ARMC5 mutation status** | | PCR for Sanger Sequencing |

## References


1. Osswald A, Quinkler M, Di Dalmazi G, et al. Long-Term Outcome of Primary Bilateral Macronodular Adrenocortical Hyperplasia After Unilateral Adrenalectomy. *The Journal of clinical endocrinology and metabolism*. 2019;104(7):2985-2993.
